# Is minimally invasive multi-vessel off-pump coronary surgery as safe and effective as MIDCAB?

**DOI:** 10.3389/fcvm.2024.1385108

**Published:** 2024-08-29

**Authors:** Magdalena I. Rufa, Adrian Ursulescu, Juergen Dippon, Dincer Aktuerk, Ragi Nagib, Marc Albert, Ulrich F. W. Franke

**Affiliations:** ^1^ Department of Cardiovascular Surgery, Robert Bosch Hospital, Stuttgart, Germany; ^2^ Institute for Stochastic and Applications, Stuttgart University, Stuttgart, Germany; ^3^ Department of Cardiothoracic Surgery, Barts Heart Centre, St. Bartholomew’s Hospital, London, United Kingdom

**Keywords:** coronary artery bypass grafting (CABG), minimally invasive off-pump coronary artery bypass grafting (MICS CABG), minimally invasive direct coronary artery bypass grafting (MIDCAB), coronary artery disease (CAD), off pump coronary artery bypass (OPCAB)

## Abstract

**Introduction:**

The safety and efficacy of minimally invasive direct coronary artery bypass (MIDCAB) surgery has been confirmed in numerous reports. However, minimally invasive multi-vessel off-pump coronary artery bypass grafting (MICS CABG) has lower uptake and has not yet gained widespread adoption. The study aimed to investigate the non-inferiority of MICS CABG to MIDCAB in long-term follow-up for several clinical outcomes, including angina pectoris, major adverse cardiac and cerebrovascular events (MACCE) and overall survival.

**Methods:**

This is an observational, retrospective, single center study of 1,149 patients who underwent either MIDCAB (*n* = 626) or MICS CABG (*n* = 523) at our institution between 2007 and 2018. The left internal thoracic artery and portions of the radial artery and saphenous vein were used for the patients’ single-, double-, or triple-vessel revascularization procedures. We used gradient boosted propensity-score estimation to account for possible interactions between variables. After propensity-score adjustment, the two groups were similar in terms of preoperative demographics and risk profile. Long-term follow-up (mean 5.87, median 5.6 years) was available for 1,089 patients (94.8%).

**Results:**

A total of 626, 454 and 69 patients underwent single, double and triple coronary revascularization, respectively. The long-term outcomes of freedom from angina pectoris, acute myocardial infarction, and revascularization rate were similar between the two groups. During follow-up, there were 123 deaths in the MIDCAB group and 96 in the MICS CABG group. The 1-, 3-, 5-, and 10-year survival rates were 97%, 92%, 85%, and 69% for the MIDCAB group and 97%, 93%, 89%, and 74% for the MICS CABG group, respectively. The hazard ratio of overall survival for patients with two or more bypass grafts compared to those with one bypass graft was 1.190 (*p*-value = 0.234, 95% CI: 0.893–1.586). This indicates that there was no significant difference in survival between the two groups. Furthermore, if we consider a hazard ratio of 1.2 to be clinically non-relevant, surgery with two or more grafts was significantly non-inferior to surgery with just one graft (*p*-value = 0.0057).

**Conclusion:**

In experienced hands, MICS CABG is a safe and effective procedure. Survival and durability are comparable with MIDCAB.

## Introduction

Coronary artery bypass grafting (CABG) is a surgical procedure that provides long-term benefits to patients with advanced coronary artery disease.

The traditional CABG surgery, which involves a median sternotomy and the use of cardiopulmonary bypass (CPB) with or without cardioplegic solution, is invasive and carries the risk of complications.

Therefore, various minimally invasive CABG techniques are being developed to reduce the invasiveness of the procedure while maintaining its benefits (1). Minimally invasive direct coronary artery bypass grafting (MIDCAB) is the most common type of minimally invasive CABG. It has been safely and effectively performed for over 20 years (2–6). MIDCAB was originally developed as an alternative to conventional CABG for patients with isolated proximal left anterior descending (LAD) artery stenosis who were not suitable candidates for percutaneous coronary intervention (PCI). In addition to its original purpose of revascularizing the LAD using the left internal thoracic artery (LITA), MIDCAB can also be used as a component of hybrid procedures for patients with multivessel disease.

Minimally invasive multivessel off-pump coronary artery bypass grafting (MICS CABG) is a more advanced type of minimally invasive CABG that can be used to bypass multiple blocked arteries in the heart, including those in the anterior, posterior, and lateral walls. MICS CABG is performed through a slightly larger incision in the left chest wall than MIDCAB. MICS CABG indications are similar to those of conventional CABG with median sternotomy.

Despite showing very good results (1, 7–9), MICS CABG has a slow adoption rate on a large scale. Careful patient selection is essential for effective minimally invasive coronary revascularization. Current contraindications for the technique include severe left pleural fibrosis and adhesions, severe pectus excavatum, advanced pulmonary disease, morbid obesity, severe left ventricular dysfunction, and emergency situations. Typically, diffuse and intramyocardial vessels are considered incompatible with MICS CABG (10).

The aim of this study was to investigate the non-inferiority of MICS CABG compared to MIDCAB in long-term follow-up for several clinical outcome variables, such as angina pectoris, major adverse cardiac and cerebrovascular events (MACCE), and overall survival.

This study is unique because it compares two minimally invasive CABG methods, both of which are underrepresented in the guidelines, in a high-volume off-pump setting. Although prospective validation is required, conducting such a study is extremely challenging when patient preference must be considered at all times.

## Methods

### Patients, data collection and study design

From January 2007 to December 2018, we performed 1,149 isolated minimally invasive off-pump CABG procedures at our facility.

Inclusion criteria for minimally invasive single or multivessel coronary artery bypass surgery at our facility include significant stenosis over 75% of the coronary artery, a maximum of three bypasses planned, favourable coronary morphology, sufficient coronary artery diameter, and a coronary artery that is easily reachable distally.

This strategy was employed in both elective and urgent situations (the latter being defined as necessitating surgery during the current hospital stay).

The exclusion criteria include emergency surgery (defined as a procedure performed prior to the commencement of the following working day after the decision to operate), obesity, small vascular disease, significant pleural adhesions of the left lung to the thoracic wall, and contraindications for single lung ventilation.

The primary objective of our study was to determine whether the number of bypass grafts had a causal effect on long-term clinical outcomes, including angina pectoris, MACCE, repeat revascularization by intervention or surgery, and overall survival. We systematically collected and analyzed adverse outcomes, clinical profiles, and demographic data. If a participant could not be contacted, we contacted their referring cardiologists or general practitioners to obtain follow-up information. The University of Tübingen's ethics committee reviewed and approved the study plan, which complied with the principles outlined in the Declaration of Helsinki (ethics registration number: 777/2021B02 from 06.12.2021). Prior to their inclusion in the research, all study participants gave their written informed consent. The corresponding author had full access to all the data in the study and takes responsibility for its integrity and the data analysis.

### Surgical technique

MIDCAB: The surgical procedure involved a 6- to 7-cm left anterior thoracotomy through the bed of the fifth intercostal space at the level of the inframammary fold. This approach facilitates easier access to the LAD. Single-lung ventilation was used to harvest the LITA under direct visualization. The LITA graft was prepared for bypass surgery with internally applied papaverine solution and systemic heparinization. A coronary shunt, vessel loops, or circular 4–0 tourniquet sutures were used for proximal occlusion of the LAD to reduce coronary backflow and avoid myocardial ischemia. A blower mister was used to stop any remaining bleeding and improve the visibility of the coronary artery anastomosis. The bypass anastomosis was performed with 8–0 polypropylene sutures. An intraoperative ultrasonic flow assessment was performed to verify the efficacy of the bypass. An intercostal nerve blockade catheter was placed to reduce postoperative pain. Patients received 500 mg of aspirin postoperatively and were typically extubated before leaving the operating room.

MICS CABG: Anterolateral access to the heart is more challenging in patients with multivessel disease, especially when multiple coronary anastomoses are required. The radial artery or saphenous vein was harvested endoscopically. The LITA was harvested and used to revascularize the LAD and sometimes the diagonal branch using a minimally invasive approach, as described above for the MIDCAB procedure. A T-graft construct was used to connect bypass grafts from the radial artery or saphenous vein to the LITA to supply blood flow to the lateral and/or posterior myocardial regions. Vacuum stabilization devices were used to maintain stability during the anastomotic procedure. The anastomoses were performed with 8–0 polypropylene sutures. The intraoperative course following completion of the bypasses is identical to that outlined for the MIDCAB procedure.

### Statistical analysis

Categorical variables are presented as a number and percentage of patients for baseline characteristics. Because most numeric variables in this study aren’t normally distributed, their median and interquartile range (IQR) are given. Because this is an observational study, it was expected that the groups being compared would differ in many preoperative characteristics, such as sex, age, body mass index (BMI), EuroSCORE II, prior myocardial infarction, history of diabetes, peripheral vascular disease, atrial fibrillation, chronic obstructive pulmonary disease (COPD), arterial hypertension, renal insufficiency, carotid stenosis, left ventricular ejection fraction, and emergency status.

**Figure 1:**
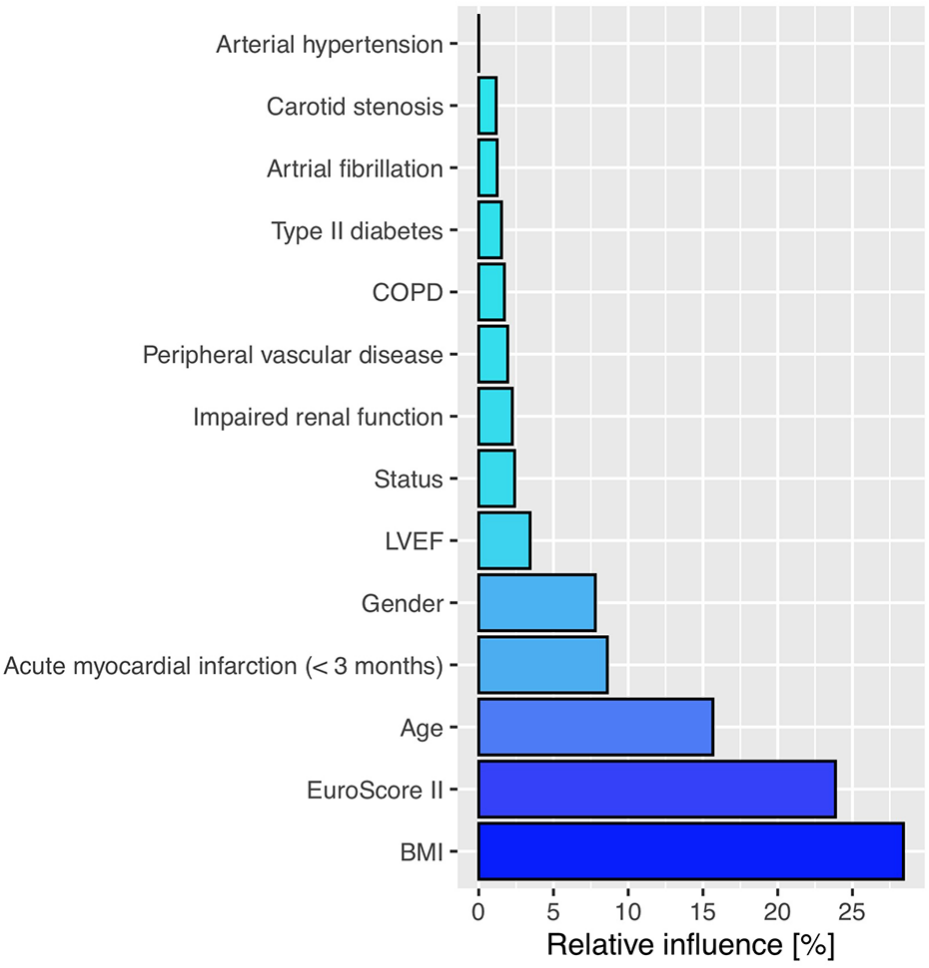
The relative influence of pre-treatment variables on the propensity score estimation. BMI, body mass index; COPD, chronic obstructive pulmonary disease; LVEF, left ventricular ejection fraction.

To allow for valid causal conclusions, we weighted each case with the reciprocal of the estimated probability this case may be found in the observed treatment group. These probabilities, the propensity scores (PS), were estimated by gradient boosted logistic regression, also allowing for possible interaction and non linearity of variables (11). Figure 1 represents the relative influence of pre-treatment variables on the PS. Appropriate group balancing was checked by considering the standardized mean differences (SMD) as an effect size measure, weighted by the propensity scores. Survival curves of right censored observations were compared by PS-weighted log-rank tests. Since MACCE-free survival data contain both left and right censored observations, a PS-adjusted interval-censored approach was undertaken (12). For all survival analyses we made use of the return date of the questionnaire. To compute the PS-adjusted *p*-value in the comparison of MACCE-free survival curves a bootstrap-type method was applied where the treatment variable was sampled according to each case's PS.

Sixty cases were lost to follow-up, and the lacking survival or MACCE data for those cases were disregarded. With the exception of this information, all other variable sets collected were entirely complete.

In order to test for non-inferiority, we considered a hazard ratio of 1.2 as being clinically non-relevant. A *p*-value of *p* < 0.05 was considered to be statistically significant. For statistical computations we used the software R, version 4.2 (13). Propensity scores were computed using the R-library twang (11). Most of the survival related analyses were undertaken using the R-libraries survival (14) and survey (15). To fit multinomial regression with weights we made use of svyVGAM (16). The interval library was employed to compare survival curves of interval censored data (12).

## Results

### Demographic data

The study population was not normally distributed. The demographic and preoperative characteristics of the study population are presented in Table 1. Patient ages ranged from 32 to 93 years (median 69, IQR 60–76) and 84.8% were male. The mean EuroSCORE II of the study participants was high, with a mean value of 5.31 (median 4, IQR 2–7). In up to three months prior to surgery, 22.9% of the patients had experienced an acute myocardial infarction, and 32.9% had already undergone percutaneous coronary intervention. The proportion of patients with a history of PCI was considerably higher in the MIDCAB group, at 37.7%. Most patients presented with normal LV EF and underwent elective procedures. 33.6% of surgeries were considered urgent. Among the 301 patients who presented with 1 VD, 248 (39.6%) underwent a MIDCAB surgery.

**Table 1:** Baseline patient characteristics.

Variable	All (1,149)	MIDCAB (626)	MICS CABG (523)	SMD	Unadj. P	Adj. SMD	Adj.P
Age (y)	69,60–76	69,59–76.7	69,61–76	0	0.491	0	0.974
Male gender	975,84.9	491,78.4	484,92.5	0.394	< 0.001	0.115	0.084
BMI	26.3, 24.2–28.7	26.3, 24.1–29	26.3, 24.3–28.4	0	0.477	0	0.726
EuroSCORE II	4 2–7	3.99 1.825–7	4 2.28–7	0	0.201	0	0.977
COPD	83, 7.2	57, 9.1	26, 5	−0.16	0.007	0.077	0.196
Smoking	245, 21.3	150, 24	95, 18.2	−0.142	0.017	−0.111	0.072
Arterial hypertension	1,142, 99.4	620, 99	522, 99.8	0.099	0.096	0.065	0.201
Atrial fibrillation	176, 15.3	102, 16.2	74, 14.1	0	0.312	0	0.529
Dyslipidaemia	1,137, 99	618, 98.7	519, 99.2	0.05	0.395	−0.027	0.632
Peripheral vascular disease	132, 11.5	85, 13.6	47, 9	−0.144	0.015	−0.066	0.287
Type II diabetes	245, 21.3	146, 23.3	99, 18.9	−0.107	0.071	−0.06	0.327
Creatinine (mg/dl)	1 0.9–1.1	1 0.8–1.1	1 0.9–1.1	0	0.068	0	0.099
Impaired renal function (GFR < 50 ml/min/1.73m^2^)	179, 15.6	110, 17.6	69, 13.2	0.121	0.042	−0.059	0.353
Renal replacement therapy	13, 1.1	9, 1.4	4, 0.8	−0.064	0.283	−0.028	0.716
Carotid stenosis	127, 11.1	69, 11	58, 11.1	0.002	0.971	−0.007	0.917
History of stroke	68, 5.9	46, 7.3	22, 4.2	−0.133	0.025	−0.123	0.043
History of MI							
• MI < 48h	14, 1.2	11, 1.8	3, 0.6	−0.108	<0.001	−0.066	0.517
• MI 2d-21d	156, 13.6	102, 16.3	54, 10.3	−0.174		−0.043	
• MI 21d-91d	93, 8.1	55, 8.8	38, 7.3	−0.056		−0.043	
• MI > 91d	151, 13.1	93, 14.9	58, 11.1	−0.111		−0.030	
History of PCI	378, 32.9	236, 37.7	142, 27.2	−0.225	<0.001	0.222	<0.001
Extent of CAD							
• 1 VD	301, 26.2	248, 39.6	53, 10.1	−0.671	<0.001	−0.671	<0.001
• 2 VD	495, 43.1	202, 32.2	293, 56	0.480		0.438	
• 3 VD	353, 30.7	176, 28.1	177, 33.8	0.124		0.169	
LV-EF:							
• 55%	899, 78.2	470, 75.1	429, 82	0.168	0.001	0.081	0.256
• 30–50%	205, 17.8	121, 19.3	84, 16.1	−0.085		−0.044	
• <30%	45, 3.9	35, 5.6	10, 1.9	−0.190		−0.086	
Status:							
• Elective	763, 66.4	442, 70.6	321, 61.4	−0.195	0.001	−0.052	0.404
• Urgent	386, 33.6	184, 29.4	202, 38.6	0.195		0.052	

Values are presented as number and percentage for categorical variables and as median and interquartile range (1Q—3Q) for continuous variables; adj. P, adjusted *P*-value; adj. SMS, adjusted standardised mean difference; BMI, body mass index; COPD, chronic pulmonary disease; EuroSCORE, European system for cardiac operative risk evaluation; GFR, glomerular filtration rate; LVEF, left ventricular ejection fraction; MI, myocardial infarction; MIDCAB, minimally invasive direct coronary artery bypass; MICS-CABG, minimally invasive multi-vessel off-pump coronary artery bypass grafting; PCI, percutaneous coronary intervention; unadj., unadjusted; VD, vessel disease.

### Perioperative outcomes

In 83.9% patients, complete revascularization was achieved, which was defined as follows: each major area of the heart that was served by a coronary artery 1.5 mm or larger with a stenosis of more than 70% or a fractional flow reserve <0.8 was addressed. However, this percentage was higher in the MICS CABG (89.9%) than in the MIDCAB (78.9%) group.

In total 142 cases, 98 (15.7%) in the MIDCAB group and 44 (8.4%) in the MICS CABG group were planned as hybrid procedures.

Hemodynamic instability and LITA injury in one case, and poor exposure as well as hemodynamic instability and intolerance to one lung ventilation in other three cases were the reasons for conversion.

All 626 MIDCAB patients and 486 (93%) of the 523 MICS CABG patients underwent total arterial revascularization. 37 patients received a saphenous vein transplant. All patients underwent coronary surgery using an aortic no-touch technique. No difference was observed in the transfusion rates, as well as intensive care unit- and hospital length of stay or in the rates of other in-hospital complications.

Perioperative death was defined as death within 30 days of operation from any cause. In total fourteen patients died in the first 30 days, 12 of them having received a MIDCAB procedure. Patients having a history of MI within the last 90 days were more common in the MIDCAB group (26.9% vs. 18.2%), as were female patients (21.6% vs. 7.5%), patients with peripheral vascular disease (13.6% vs. 9%), patients with type II diabetes (23.3% vs. 18.9%), and patients with impaired renal function (17.6% vs. 13.2%). The percentage of planned hybrid procedures (15.7% vs. 8.4%) and procedures that were initially intended as partial revascularizations (7.2% vs. 1.7%) was greater in the MIDCAB group. Additionally, in the postoperative period, the MIDCAB group had greater rates of postoperative stroke (1.8% vs. 0.4%) and new-onset renal failure requiring dialysis (2.1% vs. 1.1%). The increased 30-day mortality rate (1.9% vs. 0.4%) in this cohort could have been influenced by all of these factors.

Table 2 offers a detailed presentation of the operative and in-hospital postoperative data.

**Table 2:** Operative and in –hospital post-surgery data.

Variable	All (1,149)	MIDCAB (626)	MICS CABG (523)	SMD	Unadj. P	Adj. SMD	Adj.P
Conversion to sternotomy	4, 0.3	1, 0.2	3, 0.6	0.070	0.236	−0.068	0.365
RBC Transfusion	0, 0–0	0, 0–0	0, 0–0	0	0.081	0	0.387
FFP Transfusion	0, 0–0	0, 0–0	0, 0–0	0	0.834	0	0.936
Platelet Transfusion	0, 0–0	0, 0–0	0, 0–0	0	0.727	0	0.767
Length of ICU stay (d)	1, 1–1	1, 1–1	1, 1–1	0	0.075	0	0.084
Length of hospital stay (d)	7, 6–9	7, 6–9	7, 6–8	0	0.434	0	0.754
Postoperative new onset atrial fibrillation	14, 1.2	9, 1.4	5, 0.9	−0.044	0.442	−0.036	0.675
Postoperative new onset renal failure requiring dialysis	19, 1.7	13, 2.1	6, 1.1	−0.073	0.219	−0.054	0.454
Stroke	13, 1.1	11, 1.8	2, 0.4	−0.130	0.028	−0.093	0.271
Surgical site wound infection	28, 2.4	19, 3	9, 1.7	−0.085	0.151	−0.049	0.464
Postoperative CPR	11, 1	8, 1.3	3, 0.6	−0.072	0.223	−0.078	0.145
Postoperative MI	11, 1	6, 1	5, 1	0	0.997	−0.019	0.775
Reoperation for bleeding	33, 2.9	21, 3.4	12, 2.3	−0.063	0.284	−0.058	0.319
Reoperation with bypass revision	7, 0.6	3, 0.5	4, 0.8	0.037	0.536	0.045	0.494
Completeness of revascularization	964, 83.9	494, 78.9	470, 89.9	0.298	<0.001	0.249	<0.001
Planned as hybrid procedures	142, 12.4	98, 15.7	44, 8.4	−0.22	<0.001	−0.161	0.006
Planned as incomplete revascularization	54, 4.7	45, 7.2	9, 1.7	−0.258	<0.001	−0.205	0.001
30-d mortality	14, 1.2	12, 1.9	2, 0.4	−0.140	0.018	−0.128	0.028

Values are presented as number and percentage for categorical variables and as median and interquartile range (1Q—3Q) for continuous variables; adj. P, adjusted *P* value; adj. SMD, adjusted standardised mean difference; CPB, cardiopulmonary bypass; CPR, cardiopulmonary resuscitation; FFP, fresh frozen plasma; ICU, intensive care unit; MIDCAB, minimally invasive direct coronary artery bypass; MICS-CABG, minimally invasive multi-vessel off-pump coronary artery bypass grafting; MI, myocardial infarction; RBC, red blood cells; unadj., unadjusted.

### Survival outcomes

The mean follow up time was 5.87 years (median 5.6, IQR 3.27–8.48) and it was completed for 1,089 (584 MIDCAB, 505 MICS CABG) patients, corresponding to a follow up percentage of 94.8%. Sixty patients were lost to follow up.

The survival results did not differ significantly between the non-weighted and PS-weighted analyses. The following are the weighted results: rate of recurrent angina pectoris 91 (14.5%) vs. 75 (14.3%), *p* = 0.754, odds ratio (OR): 0.946, 95% confidence interval (CI): 0.667–1.340, myocardial infarction 30 (4.8%) vs. 20 (3.8), *p* = 0.585, OR: 0.846, 95% CI: 0.462–1.546, revascularization by means of PCI 64 (10.2%) vs. 70 (13.4%), *p* = 0.180, OR: 1.310, 95% CI: 0.885–1.913, revascularization by redo surgery 5 (0.8%) vs. 2 (0.4%), *p* = 0.575, OR: 0.603, 95% CI: 0.103–3.537 and stroke 26 (4.2%) vs. 23 (4.4%), *p* = 0.855, OR 0.945, 95% CI 0.519–1.724.

**Figure 2:**
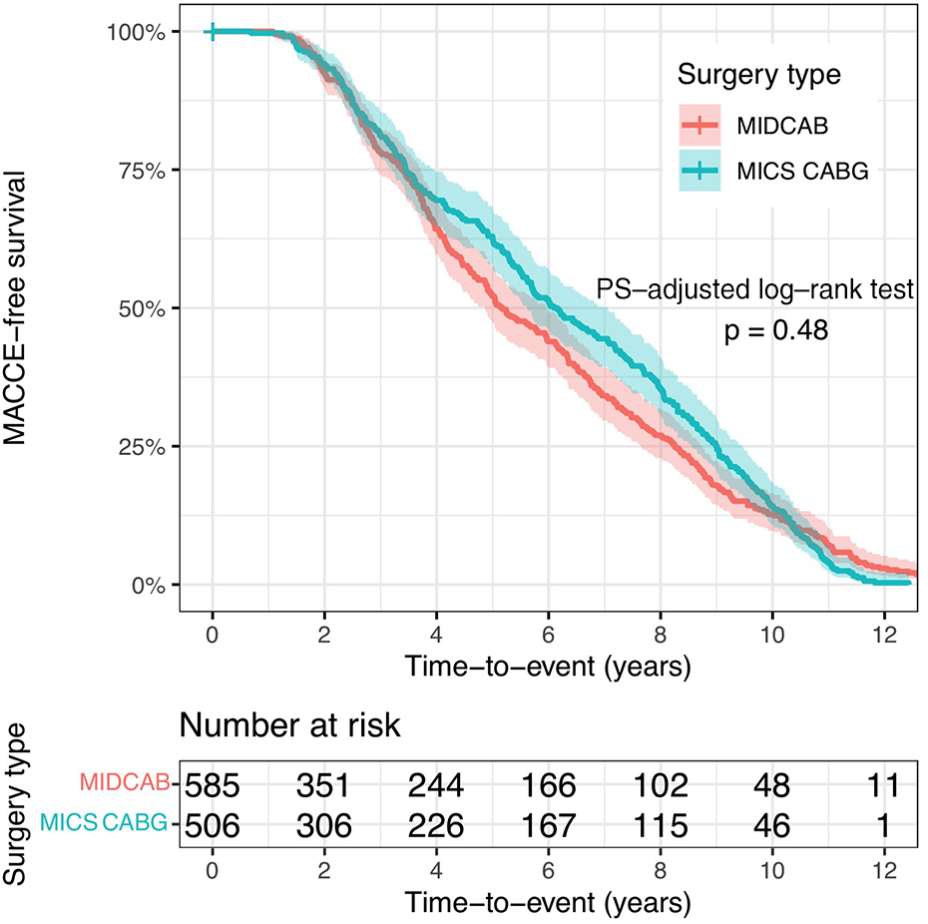
The MACCE-free survival curves for the two PS-adjusted study populations. MIDCAB, minimally invasive direct coronary artery bypass grafting; MICS CABG, minimally invasive off-pump coronary artery bypass grafting.

Additionally, there was no difference in the cumulative MACCE rate between the two groups 206 (32.9%) vs. 181 (34.6%); in the weighted analysis, *p* = 0.950, OR: 0.991, 95% CI 0.761–1.291, long rank test *p* = 0.480, and in the non-weighted analysis, *p* = 0.845, OR: 1.025, 95% CI 0.799–1.315, *p* = 0.559. Figure 2 displays the MACCE free survival curves for the two PS-adjusted research populations.

During follow-up there were 123 deaths in the MIDCAB Group and 96 in the MICS CABG Group. The 1-, 3-, 5- and 10-y- PS-adjusted survival rates were 97%, 92%, 85% and 69% for the MIDCAB group and 97%, 93%, 89% and 74% for the MICS CABG group.

When comparing cases with two or more bypass grafts vs. one, the hazard ratio of overall survival in the initial study populations, prior to PS weighting, was 0.817, 95% CI: 0.625–1.068, *p* = 0.138. Following PS weighting, the overall survival hazard ratio was 1.190, 95% CI: 0.893–1.586, *p* = 0.234. As a result, no significant difference in survival could be found.

The MIDCAB group included a higher rate of patients with incomplete revascularization, as well as a higher percentage of patients scheduled for hybrid revascularization. This would explain both the comparable rate of myocardial infarction and the long-term survival rate during follow-up. A further analysis of patients who were complete vs. incomplete revascularized revealed no difference in myocardial infarction risk during follow-up following gradient-boosted propensity score weighing (*p* = 0.284, log OR: 0.524, 95% CI: −0.434 to 1.483).

**Figure 3:**
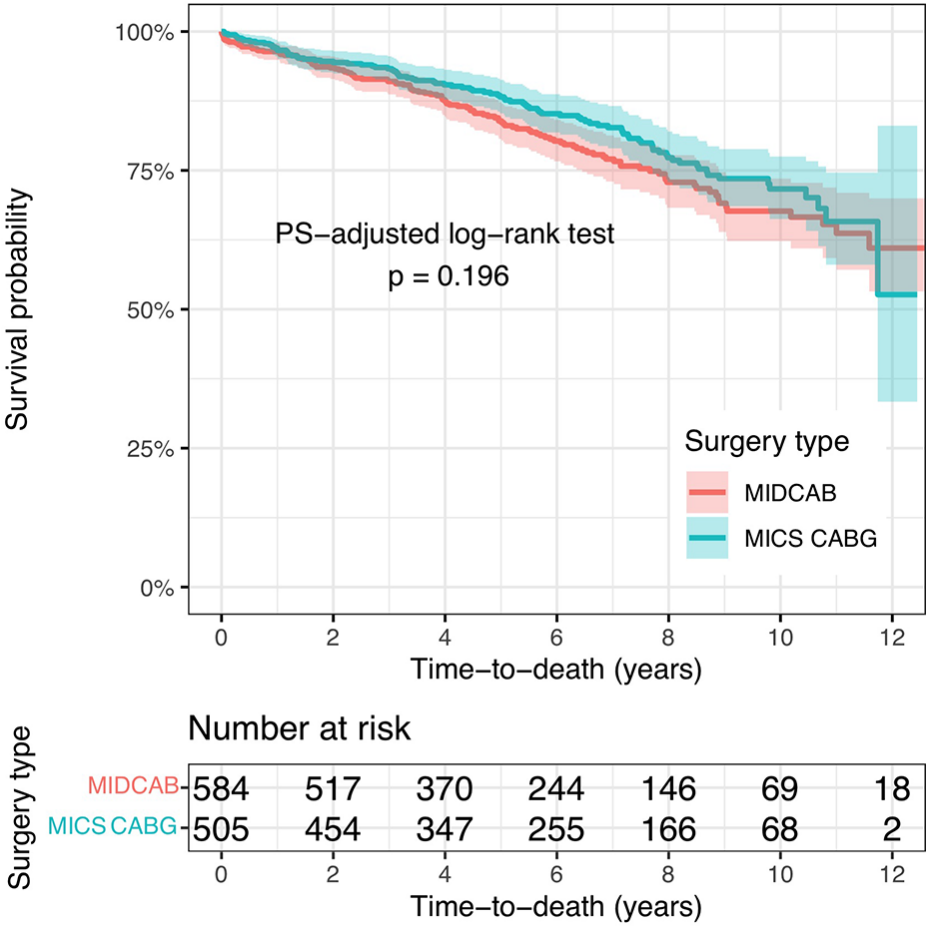
The Kaplan Meier survival curves for the two PS-adjusted study populations. MIDCAB, minimally invasive direct coronary artery bypass grafting; MICS CABG, minimally invasive off-pump coronary artery bypass grafting.

In addition, if we consider a hazard ratio of 1.2 as being clinically non-relevant, surgery with two or more grafts is significant non-inferior compared to surgery with just one graft (*p*-value 0.0057). The Kaplan Meier survival curves for the two PS-adjusted study populations are presented in Figure 3.

## Discussion

This retrospective, large-scale, single-center study compared the long-term results of single or multiple minimally invasive coronary artery bypass grafting patients on several clinical outcome variables, including angina pectoris, major adverse cardiac and cerebrovascular events, and overall survival, over a 14-year period. The study found no significant differences between the treatment groups for any of the outcome variables. The results confirm that MICS CABG is a safe and effective procedure in experienced hands, with comparable survival and durability to MIDCAB.

A growing number of patients may accept worse long-term results from surgical revascularization if they and their referring physicians view an operation as very invasive, even if it is highly successful and repeatable. Due to two large-scale randomised trials producing similar and equivocal outcomes, the argument over the best course of care for coronary artery revascularization via median sternotomy—which has been going on for more than 20 years—has not yet been resolved. After on-pump and off-pump CABG, the 5-year survival rates and the combined result of mortality, myocardial infarction, and repeat revascularization were identical in the German Off-Pump Coronary Artery Bypass in Elderly (GOPCABE) study, which included patients 75 years of age or older (17). The Coronary Artery Bypass Graft Off or On Pump Revascularization Study (CORONARY) found that patients who underwent off-pump CABG and those who underwent on-pump CABG had similar rates of the composite outcome of death, stroke, myocardial infarction, renal failure, or repeat revascularization at 5 years of follow-up. Additionally, there was no difference in quality of life measures between the groups (18).

The development of minimally invasive coronary revascularization is an exciting prospect with the potential to offer various benefits for patients. However, guidelines, clinical trial results, patient-specific characteristics, and opinions must be considered and balanced during the decision-making process in the heart team. Therefore, decision-making often risks being based on subjective judgment, with its inherent bias, rather than the strongest available evidence. Van den Eynde and colleagues propose a decision tree to guide the optimal use of minimally invasive revascularization strategies (19).

MIDCAB grafting is the most standardized minimally invasive coronary surgery. Some of its benefits include avoiding cardiopulmonary bypass and using the left internal thoracic artery, which has been shown to have excellent long-term patency (20–23). Early mortality rates in MIDCAB studies range from 0% to 4.9%, conversion rates to sternotomy are as high as 6.2%, and perioperative complication rates range from 1.6% to 40% (3, 4, 24–27). Twenty years after their first trial, Raja et al. found that MIDCAB patients in their retrospective single-center study had a 5-year survival rate of 93.6%, a 10-year survival rate of 76.2%, and a 15-year survival rate of 67.5% (2). Holzhey et al. reported a 5-year survival rate of 88.3% and a 10-year survival rate of 76.6% in their 13-year single-center experience with MIDCAB (3). Repossini et al. reported on their experience with 1,060 MIDCAB patients over 20 years. They achieved excellent outcomes, with a 5-year survival rate of 87.1%, a 10-year survival rate of 84.3%, and a 15-year survival rate of 79.8%, and with low postoperative morbidity and mortality (6). Our MIDCAB population's in-hospital outcomes are comparable to those described. The higher median EuroSCORE II of 3.99 in our MIDCAB study sample may explain the somewhat decreased survival rate in long-term follow-up.

MICS CABG, in contrast to MIDCAB, allows for multivessel coronary grafting. Rodriguez et al. demonstrated that MICS CABG can be safely initiated as a minimally invasive, multivessel alternative to open surgical coronary revascularization with outstanding mid-term outcomes in certain patients (1).

In experienced facilities, MICS CABG has been shown to be a safe alternative to sternotomy CABG in terms of early and long-term postoperative mortality and morbidity. In 2009, McGinn and Ruel reported the safety and feasibility of MICS CABG based on their dual-center experience with 450 patients who underwent the procedure in a prospective study (7). 95% of patients were successfully revascularized with a mean of 2.1 ± 0.7 implants. The perioperative mortality rate was 1.3%, 3.8% of patients required conversion to sternotomy, 7.6% required cardiopulmonary bypass, and the average duration of hospital stay was 5.9 ± 3.4 days. At a mean follow-up of 19.2 ± 9.4 months, graft stenosis was observed in 1.6% of grafts anastomosed directly on the aorta and in 5.6% of LITA T-grafts; 3% of patients required revascularization by PCI (7).

The MICS CABG Patency Study, which prospectively enrolled 91 patients from these two centers, subsequently evaluated the angiographic patency of grafts following MICS CABG (28). The median number of grafts in 89 patients who received MICS CABG was 3, and complete revascularization was achieved in all patients (100%). 76% of patients had an off-pump procedure performed (28). There was no perioperative mortality observed. The median duration of hospital stay was four days, and there were no deaths or major adverse cardiac events at six-month follow-up. At 6 months follow-up, 64-slice CT angiography was used to assess graft patency, and the overall graft patency was 92%, with 100% patency for LITA grafts and 85% for saphenous vein grafts (28).

MICS CABG has never been directly compared to MIDCAB, but several studies have compared it to sternotomy off-pump coronary artery bypass grafting (OPCAB) (29).

There are limited long-term clinical data on MICS CABG, but several observational studies have demonstrated promising clinical outcomes. Perioperative mortality rates range from 0% to 1.3%, perioperative stroke rates range from 0% to 0.4%, and conversion rates range from 0% to 6.7% (1, 7–9, 30). Studies have also demonstrated a decrease in transfusions and surgical site infection rates with MICS CABG, as well as a decrease in hospital length of stay and an earlier return to full physical function when compared to OPCAB (7, 9, 30–32).

Rabindranauth et al. found no significant difference in long-term survival or composite end point of death between MICS CABG (mean follow-up of 18.5 ± 11.5 months) and OPCAB (mean follow-up of 45.0 ± 27.8 months) (30).

Nambiar et al. demonstrated excellent outcomes for MICS CABG using bilateral internal thoracic arteries in 940 patients, with 97.9% complete revascularization, 0.9% mortality, and 99.3% freedom from major adverse cardiac and cerebrovascular events at an average follow-up of 33 months (8). Ten (1.06%) of the group's patients required reintervention, with two of those patients experiencing LITA-RITA Y anastomosis blockage (8). Their analysis found a significant reduction in death, stroke, postoperative myocardial infarction, and reintervention rates, as well as an all-cause risk reduction, when compared to both arms of the SYNTAX trial at 30 days, 12 months, and 5 years (8).

The only randomized controlled trial (STET trial) conducted to date with 184 patients failed to confirm the clinical benefits of MICS CABG demonstrated in observational studies (33). Although patients in the MICS CABG group had fewer proinflammatory cytokines, shorter intubation times (256 vs. 321 min), and fewer postoperative arrhythmias (23% vs. 35%) than those in the OPCAB group, the MICS CABG group had prolonged median hospital length of stay (6 vs. 5 days) and median operative times (4.1 vs. 3.3 h) (33). At three and twelve months, the pain and quality of life scores of both groups were comparable (33). Others have hypothesized that the precipitous learning curve of multivessel MICS CABG may have influenced the trial's outcome (34).

The Minimally Invasive coronary surgery compared to STernotomy coronary artery bypass grafting (MIST Trial) is currently enrolling participants to evaluate quality of life and MACCE in a randomized trial comparing MICS CABG to conventional CABG (35). The estimated primary completion date is March 1, 2024, with the estimated study completion date being two years later, on March 1, 2026.

The primary benefit of MICS CABG over alternative procedures is multiarterial vessel grafting employing a single internal thoracic artery or both internal thoracic arteries and/or radial artery as graft material, without the risk of sternal wound infection. We performed off-pump MICS CABG in all cases. However, most MICS CABG studies report results of aortic touch cases (29). Also, the rate of total arterial revascularization in our study population was very high, 93%, with only 37 patients receiving a saphenous vein graft.

The aforementioned MICS CABG studies report follow-up periods of up to two years; thus, to the best of our knowledge, our data represent the largest single-center MICS CABG population followed for over fourteen years.

The low rate of hospital complications in our cohort population must be highlighted. The excellent outcomes in our study are a reflection of the experience of the surgeons performing both procedures. Through the presentation of data on more than a thousand patients, we hope to persuade our peers to adopt these minimally invasive techniques more broadly, as their penetrability is still relatively low in the cardiac surgical community. Large medical centers have a true opportunity to build certified programs focused on minimally invasive cardiac surgery.

This study includes patients from 2007 to 2018. Our institution acquired the da Vinci Xi (Intuitive Surgical Inc., Sunnyvale, CA, USA) surgical robot in 2019 and the surgical team first underwent extensive training. Starting in July 2019, we began performing robotically assisted MIDCAB and MICS CABG procedures using single or bilateral internal thoracic artery as graft material. The robotic platform offers excellent visualisation of the internal thoracic arteries, minimising the risk of vessel injury. It is also less traumatic for patients compared to traditional harvesting methods. As a result, longer ITA grafts can be obtained. We anticipate that this change in technique and graft material will further enhance our outcomes. So far, we have received positive feedback from our referring cardiologists and patients regarding this transition.

Prospective randomized studies provide the most robust evidence; however, in order to validate the hypothesis prior to undertaking such a study, we rely on high-quality retrospective reports encompassing a relatively substantial number of patients. Prospective data is necessary for a more widespread implementation of MICS CABG, as this technique is not new but has not been adopted sufficiently. Advancements in drug eluting stents require surgeons and cardiologists to collaborate closely in order to provide patients with the most effective and least invasive treatment options, ensuring both short-term and long-term success. There is an increasing focus on a hybrid approach defined as MICS CABG with PCI, as opposed to MIDCAB and PCI. An essential prerequisite for organizing such a prospective trial would be to conduct a multicenter international study in centres with extensive experience in off-pump and minimally invasive procedures. The next step involves establishing clear and widely accepted guidelines and protocols for hybrid revascularisation, MIDCAB and MICS CABG procedures. This will ensure that everyone is on the same page and using a standardized language. Obtaining reliable data from this study would provide strong justification for the implementation and expansion of a minimally invasive CABG program in other medical centres, given the increasing demand from patients.

As a result, our data are of importance in advancing the medical community's ability to organize a prospective, randomized, multi-centric study of this nature.

### Limitations

One limitation of this study is its observational, retrospective design, which carries the potential for error in data collection. Propensity score weighted comparisons may adjust from known confounders, but not unknown confounders. An additional limitation of our study is the potential impact of selection bias, as only patients likely to benefit from MIDCAB or MICS CABG were selected. Finally, our results may not be generalizable to centers with less experience in off-pump and minimally invasive surgery, as MICS CABG is a complex procedure that requires the appropriate surgical expertise.

## Conclusions

MICS CABG is a highly desirable procedure for patients due to its minimal invasiveness, superior cosmesis, and rapid recovery. Although it is a challenging procedure for surgeons, experienced off-pump surgeons can perform it effectively with comparable long-term outcomes to MIDCAB. These data may help guide surgeons, their teams, and institutions in initiating a multivessel MICS CABG program.

## Data Availability

The original contributions presented in the study are included in the article/Supplementary Material, further inquiries can be directed to the corresponding author.
